# Tuberculosis screening during the 2015 European refugee crisis

**DOI:** 10.1186/s12889-020-8303-y

**Published:** 2020-02-07

**Authors:** Susanne Tewes, Bennet Hensen, Alexandra Jablonka, Dana Gawe, Maija Kastikainen, Christine Happle, Julia Carlens, Lars-Daniel Berthold, Frank Wacker

**Affiliations:** 1 Hannover Medical School, Institute for Diagnostic and Interventional Radiology, Carl-Neuberg-Str. 1, 30625 Hannover, Germany; 2 Department of Clinical Immunology and Rheumatology, Hannover Medical School, Carl-Neuberg Str. 1, 30625 Hannover, Germany; 3 Hannover Medical School, German Center for Infection Research, Hannover-Brunswick, Carl-Neuberg Str. 1, 30625 Hannover, Germany; 4 Local Public Health Department, Osterholz, Heimstr. 1-3, 27711 Osterholz-Scharmbeck, Germany; 5 Clinic for Paediatric Pneumology, Allergology and Neonatology, Hannover Medical School, Carl-Neuberg Str. 1, 30625 Hannover, Germany; 6 Biomedical Research in Endstage and Obstructive Lung Disease Hannover (BREATH), Member of the German Center for Lung Research (DZL), Hannover Medical School, Carl-Neuberg Str. 1, 30625 Hannover, Germany

**Keywords:** Tuberculosis, Screening, Refugee, Chest X-ray, Migration, Infection

## Abstract

**Background:**

The purpose of our study was to describe and evaluate management, performance and results of Tuberculosis (TB)-screening among refugees and asylum seekers in a rural area in Germany in 2015.

**Methods:**

Refugees or asylum seekers, staying in shared-accommodation are obligated to participate on screening chest X-ray (CXR) in order to screen for signs of potentially infectious pulmonary TB (German Protection against Infection Act and German Asylum Procedure Act). *n* = 705 individuals underwent screening chest X-ray (CXR) to detect pulmonary TB in September and October 2015 on site. One experienced radiologist interpreted and reported each CXR within 24 h after the enrollment in the screening program and results were sent to the local Public Health Department for potential further medical care. Image abnormalities suggestive for TB were defined according to established radiographic criteria such as pleural effusion, cavitation, consolidation, fibrous scarring or calcification. Only in case of TB-suggestive findings on CXR, further diagnostics were arranged (pulmonological examination, follow-up CXR, sputum culture, interferon-gamma release assay, bronchoscopy). Follow-up data was collected in collaboration with the local Public Health Department. Descriptive statistics were calculated using GraphPad Prism software.

**Results:**

*n* = 637 CXR examinations (90%) did not show abnormal findings, *n* = 54 CXR (8%) showed incidental findings, and *n* = 14 CXR (2%) were suspicious for acute TB. Of these, n = 14 individuals, eight underwent further TB diagnostics. Active TB was confirmed in one individual (0.001% of the screening cohort).

**Conclusions:**

Our cohort reflects current immigrations statistics in Europe and illustrates an overall low TB prevalence amongst individuals entering Germany in 2015. However, our findings support the improvement of diagnostic algorithms.

## Background

According to German asylum statistics, more than 1.1 million asylum requests were filed in Germany between 2015 and 2016 [1], which is almost 4-fold higher than in the time between 2013 and 2014 (approximately 230,000 asylum requests) [2, 3]. A large number of people arrived rather unexpectedly and needed immediate medical care and screening [4]. As the German health care system was comparably ill-prepared for such a large influx, organizational structures and equipment had to be arranged within a short time period. Accommodations for the arriving people were organized in sports centers, former military barracks or camps, and on-site outpatient clinics were improvised. This success depended largely on medical healthcare worker volunteers [4].

Prevention and assessment of communicable diseases among newly arrived migrants is essential to address their health needs [5–7] including easy access to diagnosis and treatment of tuberculosis (TB) [8, 9].

During the peak of immigration in 2015/2016, thus far unprecedented numbers of refugees and asylum seekers were a challenge for receiving health care systems [10]. In this context, screening for tuberculosis (TB) offered the opportunity to provide appropriate treatment of this severe infectious disease [8, 9]. Different modes of screening are available for screening for TB, such as medical examination, chest X-ray (CXR), tuberculin skin testing or interferon gamma release essay (IGRA) [11–13]. According to the German Protection against Infection Act [14] and the German Asylum Procedure Act [15], refugees or asylum seekers staying in shared-accommodation are obligated to participate in examinations to exclude communicable diseases. CXR examinations are obligatory for each person (except for children < 15 years of age and pregnant women) in order to screen for signs of potentially infectious pulmonary TB [14, 15]. While notification of confirmed, active TB cases to official authorities is mandatory in Germany, no obligation to document the number of screening participants exists, resulting in sparse information about yields and effectiveness of TB screening programs [16, 17].

Weinrich et al. reported a low yield of TB-screening in a metropolitan center in Germany 2015 with a number needed to screen of 1749 [18]. TB-prevalence ranged from 0.26 to 0.05% in a study by Herzmann et al., including screening results from 4, both rural and metropolitan centers in Germany 2015 [19] but difficulties with the arrangement of follow up examinations were reported. The need for data collection and analysis in order to improve screening algorithms has been addressed by several authors [9, 10, 16–23].

The purpose of this study was to describe and evaluate the management, performance and results of systematic CXR-based TB-screening in a shared-accommodation for refugees and asylum seekers in a rural area in Germany in September and October 2015.

## Methods

### CXR-based TB-screening

At a single shared-accommodation site in Germany in September/October 2015, *n* = 705 consecutive CXR were performed.

### Enrollment of TB-screening participants

According to paragraph 36 of the German Protection against Infection Act [14] and paragraph 62 of the German Asylum Procedure Act [15], refugees or asylum seekers staying in a shared-accommodation are obligated to participate in examinations to exclude transmittable disease [15] and therefore underwent CXR on-site. Pregnant women as well as children < 15 years of age did not undergo CXR and were therefore excluded from the analysis. 705 TB-screening participants were included in our analysis. The statutory mass screening for TB only comprises CXR. Only in case of suspicious findings, pulmonologist-consultation and further examinations were recommended. Names, age and gender were documented in the screening process. Patients’ history or blood samples were not collected within the first round of the screening program.

### Preparations

In order to guarantee high-quality medical care, an on-site outpatient clinic was provisionally constructed. The medical corps of the German army provided a digital X-ray radiography system (CXDI Canon Inc. system).

### Implementation

Radiologic technologists from our institution volunteered to acquire CXR from each screening participant on-site. CXR was performed in posterior-anterior view [24]. CXR images were send to our institution using a secure channel licensed for teleradiology.

One radiologist from our institution (26 years experience in CXR) performed image interpretation and reporting using our institutional image interpretation and archiving system Visage 7.1 (Visage imaging, Berlin). Image reporting focused on signs of potentially infectious TB based on previously defined and established radiographic criteria such as pleural effusion, cavitation, consolidation, fibrous scarring or calcification [25]. Interpretation and reporting of each CXR was performed within 24 h after the enrollment in the screening program, in order to enable prompt arrangements in case of TB-suggestive findings to limit potential transmission within the shared-accommodation.

### Follow-up in case of suspicious CXR-screening results

In case suspicious findings occurred in CXR-screening, recommendations for further diagnostic work-up were made. CXR-reports were sent to the local Public Health Department for arrangement of further medical care and archival storage. Confirmation diagnostics included: consultation by a pulmonologist and clinical examination, follow-up CXR, sputum culture and/or interferon-gamma release assay or bronchoscopy. These diagnostic steps were organized by the local Public Health Department, and the results were provided for statistical analysis.

### Data analysis

Image data and reports were extracted from the institutional image interpretation and archiving system Visage 7.1 (Visage imaging, Berlin). For the retrospective analysis of screening program results, all consecutive CXR from September and October 2015 were included. Follow-up data in case of TB-suggestive findings were obtained from the local Public Health Department. For the statistical analysis, all clinical and personal information was fully anonymized. Excel (Microsoft, Redmond, WA, USA) and GraphPad Prism version 6 (GraphPad Software Inc., USA) were used for statistical analysis and creation of graphs. Mean values ± standard deviations were calculated.

### Ethical approval

The local ethics committee of Hannover Medical School approved this study (3467–2017).

## Results

### Study population

**Fig. 1:**
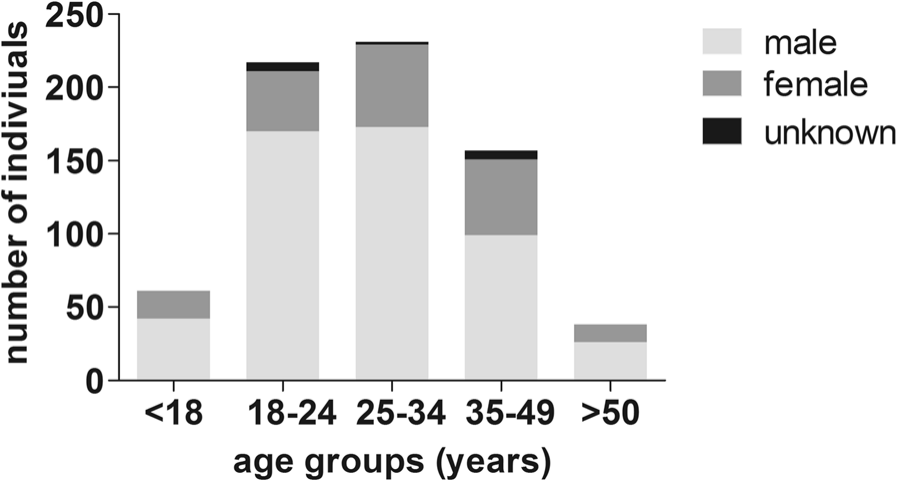
Distribution of age and gender in our study population. Depicted is the distribution of gender (in light-grey: male, in dark grey: female) divided into different age groups within our study population. In 14 individuals, no information about gender was available (in black: unknown). In one individual (male), no details were given in question of date of birth or age. This individual is not included in this graph

Out of *n* = 705 individuals, *n* = 511 were male (72.5%) and *n* = 180 were female (25.5%), and in *n* = 14 individuals no information about gender was available (2%, Fig. 1). The mean age of all individuals included into the CXR-based TB-screening analysis was 30 ± 11 years (range 15–65 years). *n* = 62 individuals (8.8%) were below the age of 18 years. In one individual, no information on the date of birth or age was available.

### CXR and follow-up

*N* = 637 CXR images (90%) did not show any abnormal findings. In *n* = 54 CXR (8%) abnormal findings not related to potentially infectious TB were found (Table 1). In *n* = 20/54 CXR, signs consistent with previous (not active) TB infection (calcified granuloma or pleural thickening) were observed. 11/54 CXR images revealed skeletal abnormalities ranging from scoliosis, additional cervical rips to untreated fractures. In *n* = 10/54 cases, mediastinal alterations were found (accentuated or enlarged shape of the heart or the upper mediastinum). In *n* = 7/54 cases, signs of unspecific inflammation were observed (infiltrates, bronchial thickening), *n* = 4/54 cases showed structural changes of the pulmonary framework (emphysema, fibrosis). In n = 2/54 CXR images, foreign bodies were identified. In *n* = 14 CXR images (2%), abnormalities suggestive for TB-infection were identified. The mean age of the subjects with TB suggestive CXR findings was 39 ± 13 years (range 17–57 years), and *n* = 11/14 were male. In these n = 14 individuals, follow-up diagnostics were initiated by local health authorities.

**Table 1:** Incidental findings on CXR, not related to acute TB-infection

54/705 CXR-screened individuals (8%)	Incidental findings
Of these individuals with incidental findings:	
20/54 (37%)	signs consistent with previous TB infection (calcified granuloma, pleural thickening)
11/54 (20,4%)	skeletal abnormalities (scoliosis, untreated fractures, cervical rips)
10/54 (18,5%)	mediastinal alterations (accentuated or enlarged shape of the heart or the upper mediastinum)
7/54 (13%)	unspecific inflammation
4/54 (7,4%)	structural changes of the pulmonary framework (emphysema, fibrosis)
2/54 (3,7%)	foreign bodies

*CXR* Chest X-ray, *TB* Tuberculosis

### Follow-up of individuals with TB-suggestive CXR

A follow-up analysis revealed that 1/14 individuals was clinically examined by a pulmonologist without signs of acute infection. In 7/14 individuals, sputum samples were smear-negative and cultures remained negative for acid-fast bacilli. In 3/7 individuals with sputum cultures, IGRAs were performed, 2/3 with negative results and 1/3 IGRA was positive. 6/14 (43%) screening participants with radiologic signs suggestive for TB were lost to follow-up.

## Discussion

With only one confirmed case of TB among *n* = 705 screening participants, the overall rate of identified TB infections in our study population was low (0.001%). For comparison, in serial CXR examinations that were performed after World War II until the late 1980s in Germany, a TB-prevalence of 0.25% was determined in the general population between 1945 and 1957, declining to 0.004% in the 1980s when serial X-ray examinations for TB-prevalence screenings were ceased [26]. The young age (Fig. 1) and good general condition in our population with only few incidental findings on CXR images (Table 1) may have contributed to this low TB-prevalence. The low rate of TB-patients identified in our screening cohort is in line with the observations by other authors: Weinrich et al. described the number needed to screen to identify a case of active pulmonary TB amongst refugees in a German metropolitan area in 2015 to be *n* = 1749 with only ten confirmed cases of active TB in *n* = 306 CXR-based screening positive individuals [18].

Another study evaluated data from four German refugee reception centers and reported differences depending on the refugees origins: among Syrian refugees, the number needed to screen to identify a case of active TB was *n* = 3000, whereas amongst refugees from Somalia, only *n* = 94 needed to be screened [19]. This illustrates that the individual risk for TB is heterogeneous among screening participants, hence, a risk group classification based on origin, medical history, age and personal environment could help to lower the number needed to screen [27] and assure that the benefit of participation in a screening program is greater than the harm of exposure to unnecessary radiation.

The mentioned study by Herzmann et al. reported an overall TB-prevalence of 140 per 100.000 screening participants. Still, one third of individuals with TB-suggestive findings in CXR imaging were lost to follow-up in their study [19]. This is supported in our study with a very low TB-prevalence but 6/14 screening participants with TB-suggestive findings on CXR lost to follow-up, strongly supporting the need for better care provision with standardized arrangement of further diagnostic work-up.

A major challenge that was reported by the Public Health Department was the language barriers. Translators were difficult to find in this rural German area, especially for certain dialects. Therefore, the need for medical procedures could not sufficiently be explained and might be a major reason why further examinations or treatment could not be initiated. Also, some screening participants were quickly transferred to a different accommodation site before screening results came back.

It is remarkable that such problems were not reported from a recent study conducted in a metropolitan German area with a large number of refugees [18] and from a centralized entry screening site in The Netherlands [28].

The main reasons for better follow-up-rates in other such as the Dutch setting could be improved administration through a centralized national reception center and lower mobility of the refugees in a metropolitan region. The procedure of migrant registration and identification, immigration interviews with national authorities and medical evaluation were completed in a standardized procedure within a time frame of 72 h upon arrival. During this standardized procedure, a team of medical technical assistants from the Public Health TB Clinic was able to take standardized questionnaires with telephone translators, to perform tuberculin skin testing, to collect sputum samples, and to immediately treat individuals with suspicion of active TB [28]. The implementation of standard-operating procedures and bundling of competencies in such a centralized entry process could improve successful implementation of TB-screening programs.

## Limitations

Besides the retrospective design of our analysis with a single center design and limited case numbers, it is a central limitation of our study that we were not able to analyze results according to region of origin, route of migration or other individual factors since these information has not been collected within the screening program but may have contributed to the personal risk for TB infection.

## Conclusions

Taken together, our data shows that TB prevalence amongst screening participants was low. In our setting, language barriers and the lack of stringent administration structures may have contributed to the high proportion of lost-to-follow-up screening participants. Our findings support the need for better care provision with standard-operating procedures. Further research and data collection is necessary to improve screening algorithms and develop risk group stratifications.

## Data Availability

Data generated or analysed during this study are included in this published article or are available in more detail from the corresponding author upon reasonable request.

## Abbreviations

CXR: Chest X-ray
IGRA: Interferon-gamma release assay
TB: Tuberculosis
